# Omega-3 Polyunsaturated Fatty Acids: Structural and Functional Effects on the Vascular Wall

**DOI:** 10.1155/2015/791978

**Published:** 2015-08-02

**Authors:** Michela Zanetti, Andrea Grillo, Pasquale Losurdo, Emiliano Panizon, Filippo Mearelli, Luigi Cattin, Rocco Barazzoni, Renzo Carretta

**Affiliations:** Department of Medicine, Surgery and Health Sciences, University of Trieste, Strada di Fiume 447, 34149 Trieste, Italy

## Abstract

Omega-3 polyunsaturated fatty acids (n-3 PUFA) consumption is associated with reduced cardiovascular disease risk. Increasing evidence demonstrating a beneficial effect of n-3 PUFA on arterial wall properties is progressively emerging. We reviewed the recent available evidence for the cardiovascular effects of n-3 PUFA focusing on structural and functional properties of the vascular wall. In experimental studies and clinical trials n-3 PUFA have shown the ability to improve arterial hemodynamics by reducing arterial stiffness, thus explaining some of its cardioprotective properties. Recent studies suggest beneficial effects of n-3 PUFA on endothelial activation, which are likely to improve vascular function. Several molecular, cellular, and physiological pathways influenced by n-3 PUFA can affect arterial wall properties and therefore interfere with the atherosclerotic process. Although the relative weight of different physiological and molecular mechanisms and the dose-response on arterial wall properties have yet to be determined, n-3 PUFA have the potential to beneficially impact arterial wall remodeling and cardiovascular outcomes by targeting arterial wall stiffening and endothelial dysfunction.

## 1. Introduction

Cardiovascular disease is the first cause of death in the developed world. Its main feature is the extensive presence of atherosclerosis, which is anticipated by morphologic and functional changes involving vessel wall and vascular endothelium. Impairment of functional properties of the arteries is strictly related to the morphologic changes in vessel structure and to the alteration in mechanical properties [1, 2]. Endothelial dysfunction is characterized by impaired endothelium-dependent vasodilation and “endothelial activation,” which is associated with a proinflammatory and procoagulatory milieu that promotes development and progression of vascular disease [3, 4]. Cardiovascular risk factors are closely linked to the development of endothelial dysfunction and arterial wall stiffness, which are significant predictors of cardiovascular risk and mortality [5, 6]. A synergistic interplay exists among the anatomic structures of the vessel wall, the vascular endothelium, endothelial-derived factors, and circulating cytokines, and such interplay promotes the development of overt atherosclerosis.

Omega-3 polyunsaturated fatty acids (n-3 PUFA) have shown the potential to beneficially impact fundamental steps involved in the development of preclinical atherosclerosis [7]. By targeting arterial stiffness and endothelial dysfunction, administration of n-3 PUFA may prevent atherosclerosis and cardiovascular disease. A wide range of molecular and physiological pathways are affected by n-3 PUFA administration and are involved in the regulation of arterial stiffness and endothelial dysfunction.

This review will focus on the complex nature of arterial stiffness and endothelial dysfunction and on the translational potential of n-3 PUFA for treating vascular remodeling.

## 2. Structural and Mechanical Properties of the Arterial Wall

Arterial wall consists of a complex morphological organization, with multiple layers designed to maintain the fundamental properties of blood carrying and blood pressure regulation. This structure is aimed at maintaining the elastic properties of the arterial wall, which are important for the physiological vascular function [8]. The distensibility of arterial vessels determines the amplitude of pulse pressure as well as the speed of the propagating pulse wave in the arterial system. The loss of elastic properties leads to arterial stiffness, a parameter that has been recognized in recent years as an intermediate endpoint for cardiovascular morbidity and mortality, independently of conventional risk factors for cardiovascular disease [9]. Arterial stiffening or arteriosclerosis, which is caused by the loss of the medial arterial load bearing components of the arterial wall, is pathologically distinct from the obstructive condition of arteries or atherosclerosis, usually defined as the deposition of lipids, white blood cells, and calcium in the arterial wall [10], although these two conditions are closely intertwined [11]. Arteriosclerosis could directly promote cardiovascular disease, by increasing pulsatile load on heart, reducing myocardial blood flow, damaging small vessels in kidney or brain, and by promoting atherogenesis through a reduction in shear stress rate. On the other hand, the presence of atherosclerotic plaques could mechanically alter the properties of the arterial wall.

The assessment of arterial stiffness has been increasingly used in clinical setting, considering its good predictive value for cardiovascular events. A large number of studies have been addressed to understand the mechanisms and factors influencing the development of arterial stiffness and to report interventions able to influence arterial wall properties [9]. Reducing arterial stiffness may be useful to reduce incidence of cardiovascular events and mortality; thus evidence-based treatments would be clinically important, but currently a specifically designed therapeutic strategy for this purpose has not yet been defined. Dietary habits are well-known determinants of the vascular changes occurring in the arterial wall with age, and many studies have focused on the effects of pharmacologic and nonpharmacologic interventions to modulate arterial elastic properties [12].

## 3. Effects of n-3 PUFA on Atherogenesis

A large body of evidence demonstrates the substantial benefits of n-3 PUFA in atherogenesis [13]. n-3 PUFA play several important roles in cellular molecular mechanisms, tissue metabolism and regulation, and act as pleiotropic agents on the cardiovascular system [14]. The mechanisms through which n-3 PUFA interfere with atherogenesis are therefore multiple. Their effect on endothelial dysfunction, oxidative stress, and inflammation, causing the onset of atherogenesis, will be discussed in the next section. The antiatherogenic effect of n-3 PUFA on serum lipid profile, with a reduction in both triglycerides and very-low-density lipoproteins, is well known and has recently been reviewed [15, 16]. However, it is unclear as to how many of cardiovascular benefits are related to n-3 PUFA lipid lowering effects and how many of them are due to lipid-independent effects.

Regarding the lipid deposition in the arterial layers, which is associated with atherogenesis [17], in a mouse animal model, n-3 PUFA were able to protect the arterial wall by decreasing the LDL uptake and by directing lipid deposition away from the aortic media, by decreasing the lipoprotein lipase expression [18]. This antiatherogenic effect is also associated with a reduction of macrophages and other proinflammatory markers and is enhanced by an incremental replacement of n-3 PUFA in the diet [19].

The proliferation of vascular smooth muscle cells and their lipid accumulation are associated with early lesion in the arterial wall and atherosclerosis promotion [20], highlighting the role of these cells in the pathophysiology of vascular remodeling [21]. An effect of n-3 PUFA on vascular smooth muscle cells activation has been reported in several studies. In culture cells, EPA and DHA were incorporated into phospholipids and slow down the progression of cell cycle, by inhibiting DNA synthesis and replication, thus suppressing vascular smooth muscle cells proliferation [22]. A similar inhibition in the proliferation of vascular smooth muscle cells was observed in human coronary arteries after consumption of fish oil, with a regulation of adhesion molecules on these cells [23].

A specific effect of n-3 PUFA on plaque stability has also been reported, in preventing the rupturing of vulnerable plaques, that leads to arterial thrombosis and obstruction. This effect could explain the reduction in cardiovascular endpoints observed in short-term trials conducted with n-3 PUFA. In patients undergoing carotid endarterectomy, atherosclerotic plaques revealed reduced macrophages infiltration and more stable morphology after n-3 PUFA administration [24]. In a more recent study, in plaques of patients supplemented with n-3 PUFA analyzed after carotid endarterectomy, reduced inflammation and significantly lower levels of mRNA for matrix metalloproteinases were observed [25].

The evaluation of intima-media thickness (IMT) has long been used as a marker of atherosclerotic involvement of arterial walls and as a surrogate endpoint of cardiovascular disease [26]. Although several observational studies reported an inverse association of n-3 PUFA administration, as diet consumption or fish oil administration, a systemic review of human intervention studies could not draw a firm conclusion on the effects of n-3 PUFA administration on IMT [27]. Also more recent trials were inconclusive: a positive effect on IMT was observed in patients with type 2 diabetes [28], although, in elderly men with hypercholesterolemia, a favorable effect on IMT progression was not confirmed, whereas n-3 PUFA imposed an improvement in arterial elasticity [29]. Recent cross-sectional studies reported that DHA levels, but not EPA, have an inverse association with IMT, suggesting that DHA may have a more potent antiatherogenic effect than EPA, independently of other risk factors [30].

## 4. Influence of n-3 PUFA on Arterial Wall Stiffening

As above mentioned, the alteration of mechanical properties of the arterial wall is strictly connected with atherosclerothic involvement. An increased plaque burden and a modification in the composition of arterial layers can hamper arterial elastic behavior. Nevertheless, although sharing some common risk factors, these two processes should be considered separately. While effects of n-3 PUFA on atherogenesis, on atherosclerotic plaques stability, and on arterial restenosis have been previously extensively reviewed, we will focus on n-3 PUFA effects on hemodynamic properties of the large arteries. Arterial stiffness, while being firstly determined by traditional risk factors for cardiovascular disease, can be influenced by passive mechanisms that consider mechanical and elastic properties of the vessels, and active mechanisms, regulated by the cellular and molecular function of the endothelium, the vascular smooth cells, and the extracellular matrix [1]. Some of these mechanisms may be influenced by n-3 PUFA intake.

Arterial blood pressure is considered the main determinant of arterial stiffness [9]. A fundamental mechanical property of the arteries is that the arterial wall becomes stiffer when the distending pressure becomes higher. Hypertension can also increase arterial stiffness chronically, by inducing elastin fragmentation and arterial wall remodeling [31]. A large body of studies demonstrated that n-3 PUFA are able to reduce systemic blood pressure [32], and a recent meta-analysis confirmed that a consumption of >2 g/d of EPA + DHA can reduce systolic and diastolic blood pressure in humans [33]. Thus, blood pressure, a main factor associated with arterial stiffening, is influenced by n-3 PUFA intake, explaining part of the beneficial effect of fatty acids on the arterial wall.

Triglyceride levels are known to be affected by n-3 PUFA intake. A supplementation of 2-3 g/d of EPA + DHA can reduce triglyceride levels by 25–30%, although a slight increase of LDL levels was observed in some studies [34]. Lipid abnormalities are well-known determinants for the development of atherosclerotic vessels disease and related abnormalities, such the stiffening of large arteries. In large cross-sectional studies [35] triglyceride levels were strongly associated with arterial properties, although a specific benefit in arterial stiffness levels with therapies targeting triglycerides has not been demonstrated yet.

Elevated heart rate has been shown to be associated with an increased risk of cardiovascular events, and there is evidence that the heart rate is independently associated with the progression of arterial stiffness, both in animal models and in humans [36]. n-3 PUFA supplementation is able to reduce resting heart rate and recovery after exercise. Experimental studies suggested that heart rate lowering could result from direct effects on cardiac electrophysiology [37, 38]. Some studies also suggested that n-3 PUFA might improve neurogenic autonomic function of cardiovascular system, through a modulation of vagal and sympathetic balance [39], and an independent association between aortic stiffness and muscle sympathetic nerve activity has been reported [40].

The effect of n-3 PUFA on classical risk factors for cardiovascular disease may explain the favorable effect on arterial stiffness. Nevertheless other mechanisms, mediated through biochemical cellular signaling and through neurogenic and neuroendocrine pathways, have been explored. The association between endothelial dysfunction and increased arterial stiffness has been demonstrated in vitro [41] and in vivo, both in animals and in humans [42, 43]. Considering the known effect of n-3 PUFA on endothelial function, which is discussed later in this review, this could be a main explanation of the reduction of arterial stiffness observed in experimental condition of n-3 PUFA supplementation. An enhancement in endothelial-dependent vasodilation of the muscular arterioles leads to a decrease in arterial stiffness because mechanical stresses are transferred to elastin components of the wall and because there is a reduction in reflected pulse waves [44]. A direct vasodilatory effect and an inhibition of constrictor response of DHA have been demonstrated in humans [45]. An interrelation of this vasodilatory effect has been found with different endocrine pathways, as the vascular constrictor response to angiotensin [46] and norepinephrine [47] is attenuated by n-3 PUFA in humans.

Therefore, the improvement in arterial properties shown after n-3 PUFA supplementation is multifactorial and involves both passive and active mechanisms of arterial hemodynamics, mediated by multiple cellular and molecular pathways and influenced by some major cardiovascular risk factors (hypertension, blood lipids, and autonomic balance).

## 5. n-3 PUFA and Arterial Stiffness: In Vivo Studies

Many studies have focused directly on the evaluation of arterial stiffness after n-3 PUFA supplementation (Table 1). Considering animal models, Sato et al. [48] found that supplementation of EPA reduced aortic PWV in high-cholesterol-diet-fed rabbits. Masson et al. [49] reported that pulse pressure obtained from telemetry, an index of arterial stiffness, was reduced by n-3 PUFA in fructose-fed rats, a model of insulin-resistant state. Similarly, Engler et al. [50] demonstrated that DHA supplementation reduced pulse pressure and vascular wall thickness in spontaneously hypertensive rats. More recently our group demonstrated that n-3 PUFA supplementation prevents arterial stiffening [51] and other vascular changes, such as baroreflex sensitivity [52] induced by ovariectomy, in a rat experimental model of menopause.

A number of randomized and controlled clinical trials have been conducted to explore the effects of n-3 PUFA on various endpoints related to arterial stiffness. A well-conducted meta-analysis by Pase et al. in 2011 [53], considering 10 intervention trials of n-3 PUFA supplementation, reported that the 2 main outcomes examined (PWV and systemic arterial compliance) were favorably affected by the intervention, thus providing strong support to the use of n-3 PUFA as an evidence-based mean to reduce arterial stiffness. The randomized clinical trials considered in this meta-analysis considered mainly high risk patients, with cardiovascular risk factors ranging from dyslipidemia, hypertension, and obesity to type 2 diabetes. More recent trials confirmed this result with the validated endpoint of carotid-femoral PWV, actually considered the gold standard measure for arterial stiffness [9]. These studies, performed in special patient population such as healthy smokers [54] and metabolic syndrome patients [55], confirmed a reduction in arterial stiffness. A large study conducted evaluating carotid-radial PWV in elderly men with hypercholesterolemia [29] and systemic arterial compliance in obese patients on a weight loss diet [56] confirmed a favorable effect in arterial stiffness. Considering innovative measurement methods of arterial stiffness, a small open-label study observed an improvement in regional aortic stiffness assessed by strain rate, using tissue Doppler imaging [57]. Three randomized clinical trials conducted with small doses of n-3 PUFA (<1.8 g/d) on healthy patients [58] and on young healthy patients with metabolic syndrome [59] or obesity [60] did not find any significant effect on arterial stiffness. In a trial evaluating patients with cardiovascular risk factors a fish oil diet was ineffective in reducing brachial-ankle pulse wave velocity, while the subsequent administration of pure EPA in the same population significantly reduced the arterial stiffness [61]. Considering cross-sectional studies, in general Japanese population, there was no relationship between serum omega-3 levels and arterial stiffness, evaluated as brachial-ankle PWV [62], while in a sample of 299 Korean men a regression analysis found a significant inverse association with total n-3 PUFA and carotid-femoral PWV [63].

Despite the few negative results in randomized clinical trials, current evidences generally agree that n-3 PUFA are effective in reducing arterial stiffness in humans. We can speculate that the negative results in these trials [58–60] are due to the small dose of active treatment or to the fact that in these trials a population with a low risk for cardiovascular disease was considered (young patients, healthy volunteers). The preferred use of n-3 PUFA only in high risk patients or in secondary prevention is supported by current guidelines and could be applied also for n-3 PUFA administration for the purpose of reducing arterial stiffness, although well-designed clinical trials considering high and low risk population are needed to support this evidence.

As arterial stiffness is a strong risk factor for cardiovascular disease, n-3 PUFA should be considered, among the wide range of cardiovascular drugs, as a safe and evidence-based choice to positively affect the mechanical properties of arterial wall. Which dose is the best for this outcome and which group of patients should be treated constitute an important area of future research.

## 6. Regulation of Endothelial Function and Endothelial Dysfunction

Classically the term “endothelial dysfunction” strictly refers to reduced endothelium-dependent vasodilation, which is notably associated with impaired bioavailability of the main endothelium-derived relaxing factor, nitric oxide (NO). In addition to promoting vasodilation, NO is a powerful antiatherosclerotic agent, since it reduces leukocyte adhesion, platelet aggregation, and smooth muscle cell proliferation [64]. In the endothelium NO is produced by the enzyme endothelial nitric oxide synthase (eNOS). Reduced nitric oxide bioavailability can be the result of either decreased production or increased scavenging. Several mechanisms, including downregulation of eNOS expression, posttranslational modifications of eNOS, inhibition of the enzyme catalytic activity, enzyme uncoupling, and circulating eNOS inhibitors result in decreased NO release and endothelial dysfunction [65, 66].

On the other hand, a number of studies have shown that reactive oxygen species (ROS), which are increased in many conditions associated with enhanced oxidative stress, determine endothelial dysfunction by quenching NO, reducing its bioavailability and leading to the formation of the highly toxic peroxynitrite [66, 67].

Perturbations of NO bioavailability are usually associated with signs of vascular inflammation and of a prothrombotic and procoagulable state [68]. Therefore, in a comprehensive sense, the term endothelial dysfunction encompasses a wide range of alterations of endothelial function preluding overt atherosclerosis.

Endothelial dysfunction is typically detected in conditions associated with vascular disease, such as hypertension, smoking, diabetes mellitus, hypercholesterolemia, and aging [69]. Clinically, endothelial dysfunction can be noninvasively assessed by measuring flow-mediated dilation (FMD), at the level of the brachial artery or of the coronary bed. This parameter allows determining the capability of the vessel to dilate in response to various stimuli (hyperemia following sphygmomanometer cuff inflation or infusion of muscarinic receptor agonists) [4, 70]. Importantly, several studies have demonstrated the prognostic value of endothelial dysfunction in terms of future cardiovascular events in both populations at low and high cardiovascular risk, its predictive value being not inferior to validated surrogate markers of vascular function [4, 71–73]. Therefore endothelial dysfunction can be considered an early marker of increased cardiovascular risk in patients with or without a previous history of cardiovascular disease.

## 7. Influence of n-3 PUFA on Endothelial Function

The mechanism by which n-3 PUFA influence endothelial function is mediated by their incorporation into biological membrane phospholipids; this allows modulation of membrane composition and fluidity. The importance of endothelial cell membrane composition has been documented by several studies (Table 2). The reason lies in the fact that endothelial cell membrane houses caveolae and lipid rafts where several receptors and signaling molecules crucial for cell function are concentrated [74]. Caveolae-associated receptor-mediated cellular signal transduction includes important pathways such as the nitric-oxide cGMP pathway, the NADPH oxidase and TNF-*α* –NF*κ*B induced cyclooxygenase-2 (COX-2) and prostaglandin E_2_ (PGE_2_) activation pathway [75, 76]. By modulating the composition of caveolae, as described for other classes of lipids [77] n-3 PUFA may exert their beneficial effects, which include increased NO production and reduced production proinflammatory mediators.

Molecular evidence of enhanced eNOS activity/expression following administration of n-3 PUFA derives from experimental studies in endothelial cells in culture or in animals. Wu et al. [78] showed that in bovine aortic endothelial cells and in eNOS knock-out mice EPA induces NO production by stimulating AMP-activated protein kinase (AMPK) induced endothelial nitric oxide synthase (eNOS) activation. Similarly, Omura et al. demonstrated that EPA stimulates eNOS activation in endothelial cells by inducing its dissociation from the inhibitory scaffolding protein caveolin [79]. Likewise, Stebbins et al. reported that DHA promotes eNOS activity by increasing the interaction between eNOS and HSP-90, which activates PKB/AKt pathway finally resulting in eNOS phosphorylation and activation [80]. Finally, n-3 PUFA can enhance eNOS activity by reducing the circulating levels of asymmetric dimethylarginine (ADMA), an endogenous inhibitor of eNOS, which is increased in conditions as hypertension, renal failure, and aging [81].

Another mechanism by which n-3 PUFA increase NO production is by directly stimulating eNOS gene and protein expression. Improved vasodilation as a result of n-3 PUFA induced upregulation of eNOS gene/protein expression has been documented in a wide series of reports considering physiological and disease animal models including menopause, atherosclerosis, and diabetes mellitus by our and other groups [82–88]. Taken together these data indicate a strong potential of n-3 PUFA to potentiate NO availability by enhancing its production via different molecular mechanisms.

In addition to increasing NO production, n-3 PUFA decrease oxidative stress. This effect is controversial, since the prooxidant activity of long-chain n-3 PUFA especially at high doses has long been debated [89]. However experimental studies conducted so far in cell culture or in vascular beds of experimental animals have shown that relatively large doses of n-3 PUFA improve endothelial function by attenuating ROS production as a result of a direct modulatory effect on the sources of ROS formation, including the enzymes NADPH oxidase and iNOS, finally resulting in reduced peroxynitrite formation [82, 83]. In retinal endothelial cells in culture exposed to high glucose ALA directly reduces ROS information and increased superoxide dismutase (SOD) activity [90, 91]. A potentiation of endogenous antioxidant enzyme concentrations in plasma as a direct effect of n-3 PUFA oral administration has also been reported also by other reports [92].

Among the contributors to endothelial dysfunction, n-3 PUFA have shown the potential to attenuate cellular and systemic inflammation. In endothelial cells in vitro n-3 PUFA attenuate NF-*κ*B activation, resulting in reduced VCAM-1 expression [90]. Additionally, n-3 PUFA exert systemic anti-inflammatory effects by raising the plasma levels of adiponectin [93] and suppressing the production of interleukin 6, interleukin 1*β*, soluble E selectin, and CRP [94]. These effects are dose-dependent, as relatively high doses of n-3 PUFA are required to achieve the anti-inflammatory effect and this cannot exclude the fact that indirectly also the triglyceride-lowering effect contributes to improved endothelial function often observed in these conditions.

## 8. n-3 PUFA and Endothelial Dysfunction: In Vivo Human Studies

**Figure 1:**
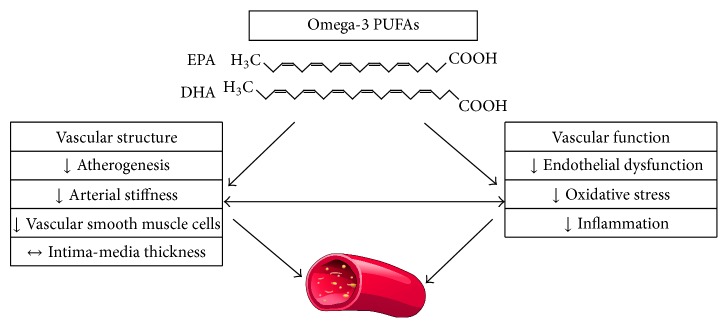
n-3 PUFA effects on vascular structure and function.

n-3 PUFA show the potential to improve endothelial dysfunction by activating NO production via different mechanisms and by reducing vascular oxidative stress and inflammation (Figure 1). Many studies have evaluated the effect of n-3 PUFA on human endothelial function and the results have been reported by two recent meta-analyses [95, 96], whose conclusions are not completely concordant. For review reasons, we will focus on studies published during the last 5 years. One issue that needs to be considered when evaluating the effect of n-3 PUFA supplementation on endothelial function is the poverty of data on n-3 PUFA basal enrichment in the patient population under consideration. The amount of n-3 PUFA in biological membranes can be directly extrapolated by measuring the omega-3 index in red blood cell membranes [97, 98] or by determining plasma concentrations of EPA + DHA which have shown a good correlation with their membrane levels [89]. This point is crucial as conditions characterized by n-3 PUFA depletion may mostly benefit from their supplementation. In a recent study conducted in an experimental model of menopause, deficiency of n-3 PUFA demonstrated by low omega-3 index was associated with endothelial dysfunction and increased oxidative stress, which were reversed by efficient n-3 PUFA supplementation, resulting in normalization of omega-3 index [82]. Having said this, clinical trials on the effects of n-3 PUFA on endothelial function are significantly heterogeneous in

- number of included participants;
- inclusion criteria: age of participants, healthy or disease state, have been studied;
- markers of endothelial function: in addition to flow-mediated dilation, at least 7 different classes of surrogate markers have been tested in the last 5 years. The most frequent categories tested, according to their different pathophysiological roles, are proinflammatory and anti-inflammatory cytokines, endothelial progenitor cells, markers of platelet activation, of fibrinolysis, of thrombosis, and of coagulation, and markers of oxidative stress [98–102];
- dose and duration of treatment: doses ranging from 0.45 up to 4 grams have been tested as well as treatments ranging from 4 to 52 weeks [58, 103, 104];
- forms of n-3 PUFA: EPA, DHA, or ALA has been administered alone or in combination;
- concomitant therapy: most of the studies on disease states do not provide accurate information on concomitant therapy, particularly on drugs known to improve endothelial function such as statins and ACE inhibitors/angiotensin receptor blockers. The presence of a robust concomitant therapy might improve endothelial function independently of n-3 PUFA (especially at low doses) in high risk patients.

So far, most studies have cautiously suggested that supplementation with n-3 PUFA might improve endothelial function. However, whether the amplitude of this effect depends on healthy or disease state or on the administered dose or whether the n-3 PUFA composition of supplementation differentially affects the outcome is currently unclear. In smokers, where the bias of concomitant therapies is not an issue, two recent studies have shown that n-3 PUFA supplementation for six and twelve weeks, respectively, improves endothelial function [54, 105]. In patients with moderate cardiovascular risk Seely et al. performed a meta-analysis where again quality and power of the available studies precluded any definite conclusion [106]. However, low-strength evidence seemed to suggest a benefit of n-3 PUFA in endothelial dysfunction. Similar results have been reported in high risk patients with previous myocardial infarction [104] although a recent comprehensive meta-analysis and a recent study in a similar population do not confirm these findings [107, 108].

When considering moderate/high risk patients assuming polytherapy, the issue of cost/benefit in terms of clinical efficiency and potential harms is important. Therefore, stronger evidence is needed before large scale prescription of n-3 PUFA in this population.

## 9. Endothelial-Independent Vasodilation and n-3 PUFA

Technically, flow-mediated dilation is the result of both endothelial-derived vasodilation (which is mainly NO-dependent) and endothelial-independent vasorelaxation. The latter depends on the ability of smooth muscle cells to respond to nitric oxide and therefore measures the integrity of arterial media. The hypotensive effect of n-3 PUFA can partly be explained by this mechanism. Therefore, when measuring FMD in vivo in humans, it is difficult to dissect the relative contribution of endothelium and smooth muscle cells unless a selective agonist is administered (muscarinic receptor agonist for EDD and NO donor for EID). A recent study addressed the physiologic mechanisms of EPA-induced relaxation in pulmonary arteries from an animal model [109] and showed that in these conditions the contribution of endothelium-derived NO release to vasodilation is prominent, while that mediated by endothelium-independent mechanisms is negligible.

These findings are in line with data from human studies, showing that when controlled trials assessing EID are considered, no significant effect of n-3 PUFA on EID is observed [96].

## 10. Conclusive Remarks

By targeting both arterial wall stiffness and endothelial dysfunction n-3 PUFA have the potential to beneficially impact arterial wall remodeling and cardiovascular outcomes. Their pleiotropic effects on systemic inflammation, modulation of lipid profile, and platelet aggregation contribute to the reduction of cardiovascular risk. Although dissecting the specific contribution of structural arterial remodeling to overall cardiovascular risk is difficult from experimental studies conducted in high risk populations, current results are encouraging. From here comes the need for large scale trials, advocated by most of the available literature. This process is likely to involve selection of homogenous patient populations in terms of target disease, endpoints, and modality of treatment.

## Figures and Tables

**Table 1a:** (a) Animal studies

First author	Year	Dose	Sample	Duration (w)	Study design	Outcome measure	Results
Sato [48]	1993	300 mg/kg/day EPA	High-cholesterol-diet-fed rabbits	12	AES-PG	cf-PWV	Prevent increase in cf-PWV

Engler [50]	2003	DHA-enriched oil	Spontaneous hypertensive rats	6	AES-PG	Wall thickness, PP	Reduce wall thickness and PP

Masson [49]	2008	diet enriched w. 16 g/kg n-3 PUFA	Fructose-fed rats	10	AES-PC	PP	Prevent increase in PP

Losurdo [51]	2014	0.65 g/kg/d versus placebo by gavage	Ovariectomized rats	8	AES-PC	cf-PWV	Prevent increase in cf-PWV

**Table 1b:** (b) Human studies

First author	Year	Dose	Sample	Duration (w)	Number	Study design	Outcome measure	Results
McVeigh [110]	1994	1800 mg EPA + 1200 mg DHA/d versus placebo (olive oil)	Type 2 diabetes	6	20	RCT-PC	Total AC	Increase in total AC

Nestel [111]	2002	3000 mg EPA/d versus 3000 mg DHA/d versus placebo (olive oil)	Dyslipidemic	7	38	RCT-PC	Total AC	Increase 36% with EPA, 27% with DHA

Tomiyama [62]	2011	1800 mg EPA/d versus control (diet therapy)	Dyslipidemic	52	84	RCT-PG	ba-PWV	Reduction of ba-PWV

Hjerkinn [29]	2006	2400 mg n-3 PUFA versus control diet	Dyslipidemic	156	563	RCT-PG	cr-PWV	Decrease in cr-PWV 4%

Hill [112]	2007	1560 mg DHA + 360 mg EPA/d versus placebo (6 g sunflower oil/d)	Overweight, hypertensive, dyslipidemic	6 to 12	38	RCT-PC	Small and large AC	Increase in small AC 26%.

Mita [28]	2007	1800 mg EPA/d versus control (no EPA)	Type 2 diabetes	6	64	RCT-PG	ba-PWV	Reduction of ba-PWV

Wang [113]	2008	540 mg EPA + 360 mg DHA versus placebo capsules	Overweight, hypertensive	8	52	RCT-PC	Small and large AC	Increase in large AC 21%

Satoh [114]	2009	1800 mg EPA/d + diet versus control (diet only)	Metabolic syndrome	12	92	RCT-PG	ca-PWV	Reduction of ca-PWV 6%

Ayer [115]	2009	32 mg EPA/d + 135 mg DHA/d + canola oil versus control diet	Healthy children	260	616	RCT-PG	Carotid artery distensibility, cb-PWV, Aix	No difference

Sjoberg [116]	2010	1560 DHA + 360 mg EPA/d versus placebo (sunola oil)	Overweight	12	67	RCT-PC	Small and large AC	Increase in large AC 14%

Dangardt [60]	2010	1200 mg n-3 PUFA versus placebo	Obese adolescents	12	25	RCT-PC	cf-PWV	No difference

Sanders [58]	2011	1800 mg n-3 PUFA versus placebo	Healthy subjects	52	312	RCT-PC	cf-PWV	Decrease in cf-PWV

Haiden [57]	2012	1800 mg n-3 PUFA versus placebo	Hypertensive, dyslipidemic	52	19	CT	ba-PWV, aortic strain rate	Decrease in ba-PWV 1%, strain rate 17%

Siasos [54]	2013	2000 mg n-3 PUFA versus placebo	Healthy smokers	12	20	RCT-PC	cf-PWV	Decrease in cf-PWV 6%

Root [59]	2013	1700 mg n-3 PUFA versus placebo	Overweight young	4	30	RCT-PC	cf-PWV	No difference

Wong [56]	2013	4000 mg n-3 PUFA + diet versus diet alone	Obese	12	13	RCT-PC	Small and large AC	Increase in large AC 20%, small AC 22%

Tousoulis [55]	2014	2000 mg n-3 PUFA versus placebo	Metabolic syndrome	12	29	RCT-PC	cf-PWV	Decrease in cf-PWV 5%

n-3 PUFA, omega-3 polyunsaturated fatty acids; EPA, eicosapentaenoic acid; DHA, docosahexaenoic acid; PWV, pulse wave velocity; cf, carotid-femoral; ba, brachial-ankle; cr, carotid-radial; PP, pulse pressure; AC, arterial compliance; AES, animal experimental study; RCT, randomized clinical trial; CT, clinical trial; PG, parallel groups; PC, placebo controlled; d, day; w, week.

**Table 2a:** (a) Animal studies

First author	Year	Dose	Sample	Duration (w)	Study design	Outcome measure	Results
Nyby [84]	2005	Diet enriched with 60% fructose and 4.4% n-3 PUFA versus diet with 60% fructose or control diet	Hyperinsulinemic rats	8	AES-PG	EDD, oxidative stress	Improve EDD and oxidative stress

Matsumoto [88]	2009	300 mg/kg/day EPA versus control diet	Diabetic rats	4	AES-PC	EDD	Improve EDD

Zhang [83]	2013	Diet enriched with ALA 500 mg/kg/day versus control diet	Type 2 diabetic rats	5	AES-PC	EDD, oxidative stress	Improve EDD and oxidative stress

Gortan Cappellari [82]	2013	800 mg/kg/day by gavage versus control diet	Ovariectomized rats	8	AES-PC	EDD, oxidative stress	Improve EDD and oxidative stress

**Table 2b:** (b) Human studies

First author	Year	Dose	Sample	Duration (w)	Number	Study design	Outcome measure	Results
Woodman [117]	2003	3800 mg EPA or 3700 mg DHA versus olive oil	Hypertensive type 2 patients	6	30	RCT-PG	EDD, EID	Unchanged EDD and EID

Engler [118]	2004	1200 mg n-3 PUFA versus control diet	Hypercholesterolemic children	10	20	RCT-PC	EDD, oxidative stress, inflammation	Improved EDD, unchanged oxidative stress and inflammation

Ros [119]	2004	1100–1700 mg n-3 PUFA versus Mediterranean diet	Hypercholesterolemic patients	4	20	RCT-PG	EDD, oxidative stress and CRP	Improved EDD, unchanged oxidative stress and inflammation

Keogh [120]	2005	4700 mg mg n-3 PUFA versus isocaloric high carbohydrate, saturated or monounsaturated fat enriched-diet	Healthy subjects	4	40	RCT-PG	EDD, CRP, inflammation	Improved EDD in all groups except in saturated fat enriched diet

Prabodh Shah [121]	2007	500 mg n-3 PUFA versus placebo	Healthy subjects	2	26	RCT-PC	EDD, EID	Improved EDD and EID

Wright [122]	2008	3000 mg n-3 PUFA versus standard therapy	Systemic lupus erythematosus patients	24	56	RCT-PG	EDD, oxidative stress	Improved EDD and oxidative stress

Schiano [123]	2008	1700–2000 mg versus standard therapy	Intermittent claudication patients	13	32	RCT-PG	EDD, inflammation	Improved EDD, inflammation unchanged

Mindrescu [124]	2008	4500 mg n-3 PUFA + rosuvastatin 10 g versus rosuvastatin 10 g	Dyslipidemic patients	4	30	RCT-PG	EDD, EID	Improved EDD and EID

Rizza [125]	2009	1700–2000 mg n-3 PUFA versus placebo	Offspring of type 2 diabetic subjects	12	50	RCT-PC	EDD, inflammation	Improved EDD and inflammation

Wong [99]	2010	4000 mg n-3 PUFA versus control (olive oil)	Type 2 diabetes mellitus	12	97	RCT-PG	EDD, CRP, renal function	Improved renal function; no effect on EDD or CRP

Stirban [100]	2010	2000 mg versus control (olive oil)	Type 2 diabetes mellitus	6	34	RCT-PC	Postprandial EDD	Improved postprandial EDD

Sanders [58]	2011	450–900 or 1800 mg n-3 PUFA versus placebo (refined oil)	Healthy subjects	51	310	RCT-PC	EDD	Unchanged EDD and EID

Skulas-Ray [101]	2011	850 or 3400 mg versus placebo	Moderate hypertriglyceridemia	8	26	RTC-PC	EDD, IL-6, CRP	No effect on EDD, IL-6, or CRP

Moertl [103]	2011	1000 or 4000 mg n-3 PUFA versus placebo	CHF	12	43	RCT-PC	LVEF, EDD, IL-6	Improved LVEF, EDD, and IL-6

Haberka [104]	2011	1000 mg n-3 PUFA versus control (standard diet and therapy)	Previous AMI	12	40	RCT-PG	EDD, EID	Improved EDD; EID unchanged

Din [105]	2013	2000 mg n-3 PUFA versus placebo	Cigarette smokers	6	20	RCT-PC	EDD, P-selectin, CD40L	Improved EDD and P selectin; CD40L unchanged

Din [108]	2013	2000 mg n-3 PUFA versus placebo	Previous AMI	6	20	RCT-PC	EDD, P-selectin, CD40L	No effect

n-3 PUFA, omega-3 polyunsaturated fatty acids; EPA, eicosapentaenoic acid; DHA, docosahexaenoic acid; EDD, endothelium dependent dilation; EID: endothelium independent dilation; AMI: acute myocardial infarction; CHF: chronic heart failure; LVEF: left ventricular ejection fraction; IL-6: interleukin-6; CRP: C reactive protein; AES, animal experimental study; RCT, randomized clinical trial; CT, clinical trial; PG, parallel groups; PC, placebo controlled; d, day; w, week.

## Abbreviations

NO: Nitric oxide
eNOS: Endothelial nitric oxide synthase
iNOS: Inducible nitric oxide synthase
NADPH: Nicotinamide adenine dinucleotide phosphate
ROS: Reactive oxygen species
TNF*α*: Tumor necrosis factor alpha
IL-1*β*: Interleukin 1 beta
IL-6: Interleukin 6
COX-2: Cyclooxygenase-2
PGE_2_: Prostaglandin E_2_
SOD: Superoxide dismutase
AMPK: AMP-activated protein kinase
EPA: Eicosapentaenoic acid
DHA: Docosahexaenoic acid
ALA: Alpha-linolenic acid
ADMA: Asymmetric dimethylarginine
PWV: Pulse wave velocity
IMT: Intima-media thickness
EDD: Endothelial-dependent dilation
EID: Endothelial-independent dilation.
